# Peginterferon alfa-2a plus Weight-Based or Flat-Dose Ribavirin for Treatment-Naïve Hepatitis C Virus Genotype 2 Rapid Responders: A Randomized Trial

**DOI:** 10.1038/srep15255

**Published:** 2015-10-15

**Authors:** Chen-Hua Liu, Chung-Feng Huang, Chun-Jen Liu, Chia-Yen Dai, Jee-Fu Huang, Jou-Wei Lin, Cheng-Chao Liang, Sheng-Shun Yang, Chih-Lin Lin, Tung-Hung Su, Hung-Chih Yang, Pei-Jer Chen, Ding-Shinn Chen, Wan-Long Chuang, Jia-Horng Kao, Ming-Lung Yu

**Affiliations:** 1 Department of Internal Medicine, National Taiwan University Hospital, Taipei, Taiwan; 2 Hepatitis Research Center, National Taiwan University Hospital, Taipei, Taiwan; 3 Graduate Institute of Clinical Medicine, National Taiwan University College of Medicine, Taipei, Taiwan; 4 Department of Internal Medicine, National Taiwan University Hospital, Yun-Lin Branch, Douliou, Taiwan; 5 Institute of Clinical Medicine and Faculty of Internal Medicine, College of Medicine, Kaohsiung Medical University, Kaohsiung, Taiwan; 6 Hepatobiliary Division, Department of Internal Medicine and Hepatitis Center, Kaohsiung Medical University Hospital, Kaohsiung, Taiwan; 7 Department of Occupational Medicine, Kaohsiung Municipal Ta-Tung Hospital, Kaohsiung, Taiwan; 8 Institute of Biomedical Sciences, National Sun Yat-Sen University, Kaohsiung, Taiwan; 9 Department of Internal Medicine, Far Eastern Memorial Hospital, Taipei, Taiwan; 10 Department of Internal Medicine, Taichung Veterans General Hospital, Taichung, Taiwan; 11 Department of Gastroenterology, Taipei City Hospital, Ren-Ai Branch, Taipei, Taiwan; 12 Department of Microbiology, National Taiwan University College of Medicine and National Taiwan University Hospital, Taipei; 13 Genomics Research Center, Academia Sinica, Taiwan

## Abstract

The impact of ribavirin (RBV) dosage on sustained virologic response (SVR) rates remains elusive in hepatitis C virus genotype 2 (HCV-2) rapid responders receiving 16 weeks of peginterferon (Peg-IFN) plus RBV. Treatment-naïve HCV-2 patients with rapid virologic response (RVR) received Peg-IFN alfa-2a 180 μg/week plus weight-based RBV (1,000 or 1,200 mg/day; cut-off body weight: 75 kg) for 6 weeks, and then randomly received Peg-IFN alfa-2a 180 μg/week plus weight-based (1,000 or 1,200 mg/day; n = 247) or flat-dose (800 mg/day; n = 246) RBV for additional 10 weeks. The primary endpoint was SVR_24_. Patients receiving weight-based and flat-dose RBV therapies had comparable SVR_24_ rates (93.5% versus 91.9%, *P* = 0.49). The risk differences (RDs) of SVR_24_ receiving weight-based and flat-dose RBV arms were 7.1% [95% CI: 0.7% to 13.6%] in males, and −5.8% [95% CI: −12.1% to 0.5%] in females (interaction *P* = 0.01). The SVR_24_ rate was higher in males receiving ≥13 mg/kg/day than those receiving <13 mg/kg/day (96.3% versus 85.1%, *P* = 0.001). In conclusion, Peg-IFN alfa-2a plus weight-based or flat-dose RBV for 16 weeks provides comparable SVR_24_ rates in treatment-naïve HCV-2 rapid responders. However, males should receive weight-based RBV to achieve a high SVR_24_ rate.

Hepatitis C virus (HCV) infection remains the leading cause of cirrhosis, hepatic decompensation, hepatocellular carcinoma (HCC) and liver transplantation^1^. While HCV genotype 2 (HCV-2) infection is relatively uncommon in North America and West Europe (except for Northern Italy), it is common in East Asia^2^,^3^,^4^. In the era of peginterferon (Peg-IFN) plus ribavirin (RBV) therapy, the SVR rates in patients with HCV-2/3 infection are higher (75–95% versus 39–79%) than those in patients with HCV-1/4 infection^5^,^6^,^7^,^8^,^9^,^10^,^11^,^12^. Although the safety and efficacy of the recently introduced sofosbuvir-based therapies are excellent for HCV-2 patients, the treatment costs and the drug availability preclude the unselected use of these agents^13^,^14^,^15^,^16^,^17^.

In HCV-2 patients who receive Peg-IFN plus RBV and who achieve rapid virologic response (RVR), the SVR rates are comparable if they receive 12–16 weeks or 24 weeks of therapy^18^,^19^. The SVR rates are also similar in these patients with different interleukin-28B (IL-28B) genotypes^20^,^21^,^22^. However, the impact of weight-based (1,000 or 1,200 mg/day) or flat-dose (800 mg/day) RBV on the SVR rates in HCV-2 patients achieving RVR remains controversial^12^,^23^,^24^. For HCV-2 patients achieving RVR after 6 weeks of induction therapy by Peg-IFN alfa-2a plus weight-based RBV, we aimed to compare the efficacy of additional 10 weeks of therapy by Peg-IFN alfa-2a plus weight-based or flat-dose RBV, and to evaluate the factors associated with anti-viral responses.

## Results

### Patient Characteristics

**Figure 1:**
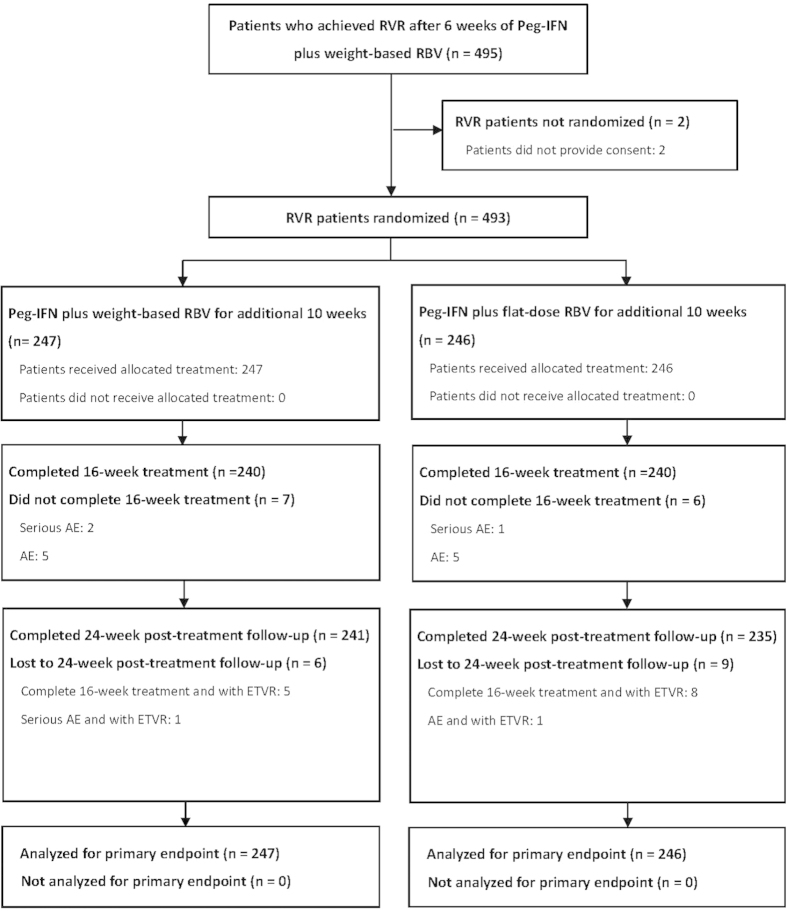
Study Flow Diagram. Peg-IFN: peginterferon, RBV: ribavirin; RVR: rapid virologic response, ETVR: end-of-treatment virologic response, AE: adverse event.

Among 495 patients who achieved RVR after 6 weeks of Peg-IFN alfa-2a plus weight-based RBV induction therapy, 493 were allocated at week 6 of treatment to receive Peg-IFN alfa-2a plus weight-based (n = 247) or flat-dosed (n = 246) RBV for additional 10 weeks. Two hundred and forty (97.2%) patients and 240 (97.6%) patients in weight-based and flat-dose RBV arms completed the assigned treatment, respectively. In addition, 234 (94.7%) patients and 230 (93.5%) patients in these two arms completed off-therapy follow-up to assess SVR_24_ (Fig. 1). The baseline patient characteristics were comparable between the two arms (Table 1). Most patients had baseline viral load of ≤800,000 IU/mL (79%) and favorable interleukin-28B (IL-28B) rs8099917 genotype (87%), and were infected with 2a subtype (75%). With regard to aspartate aminotransferase (AST) to platelet ratio index (APRI) score, 23.7% of the patients had a score of >2.00.

### Efficacy

The end-of-treatment virologic response (ETVR) (100.0% versus 99.6%, risk difference (RD): 0.4% [95% confidence interval (CI): −0.7% to 1.5%]; *P* = 0.50) and the SVR_24_ rates (93.5% versus 91.9%, RD: 1.7% [95% CI: −2.9% to 6.2%]; *P* = 0.49) were comparable between the weight-based and flat-dose RBV arms (Table 2).

We performed the sensitivity analyses to assess the impact of 6 and 9 patients who failed to achieve SVR_24_ due to undetermined reason in weight-based and flat-dose RBV arms (Table 2). As a best-case scenario, we assumed that the 6 patients in weight-based RBV arm who achieved SVR_24_ and the 9 patients in flat-dose RBV arm who failed to achieve SVR_24_. As a worst-case scenario, we assumed that the 6 patients in weight-based RBV arm who failed to achieve SVR_24_ and the 9 patients in flat-dose RBV arm who achieved SVR_24_. Results from the best-case scenario (96.0% versus 91.9%, RD: 4.1% [95% CI: −0.1% to 8.3%]; *P* = 0.10) and the worst-case scenario (93.5% versus 95.5%, RD: −2.0% [95% CI: −6.0% to 2.0%]; *P* = 0.43) were consistent with the primary endpoint.

### Subgroup Analyses for Prespecified Factors

Differences of the SVR_24_ rates between weight-based and flat-dose RBV arms did not vary by baseline viral load (interaction *P* = 0.33), subgenotype (interaction *P* = 0.61), IL-28B rs8099917 genotype (interaction *P* = 0.99), age (interaction *P* = 0.18), weight (interaction *P* = 0.71), APRI score (interaction *P* = 0.11), RBV dosage (interaction *P* = 0.88) or 80/80/80 rule (interaction *P* = 0.64) (Table 3). Compared with females (91.3% versus 97.1%, RD: −5.8% [95% CI: −12.1% to 0.5%]), males in weight-based RBV arm achieved a greater SVR_24_ rate than those in flat-dose RBV arm (95.1% versus 88.0%, RD: 7.1% [95% CI: 0.7% to 13.6%]; interaction *P* = 0.01).

We further examined the daily RBV exposure in males and females receiving weigh-based and flat-dose RBV. Compared to patients in flat-dose RBV arm, those in weight-based RBV arm received a greater RBV dosage (15.4 mg/kg/day [SD, 2.6] versus 12.9 mg/kg/day [SD, 2.1], *P* < 0.001), regardless of males (14.4 mg/kg/day [SD, 1.9] versus 12.4 mg/kg/day [SD. 2.0], *P* < 0.001) or females (16.8 mg/kg/day [SD, 2.9] versus 13.6 mg/kg/day [SD, 2.1], *P* < 0.001). Patients receiving RBV at a dosage of ≥13 mg/kg/day had a greater SVR_24_ rate than those receiving RBV at a dosage of <13 mg/kg/day (95.7% versus 87.1%, *P* = 0.001). While the SVR_24_ rates were similar in females receiving RBV at a dosage of ≥13 and <13 mg/kd/day (94.3% versus 93.9%, *P* = 0.99), the SVR_24_ rate in males receiving RBV at a dosage of ≥13 mg/kg/day was greater than those receiving RBV at a dosage of <13 mg/kg/day (97.0% versus 84.3%, *P* < 0.001) (Table 4).

### Safety

The constitutional and laboratory adverse events (AEs) were similar between two arms (Table 5). Two patients in weight-based RBV arm and one in flat-dose RBV arm had serious AEs during treatment (0.8% versus 0.4%, RD: 0.4% [95% CI: −1.0% to 1.8%]). The AE-related withdrawal rates were 2.8% in weight-based RBV arm and 2.4% in flat-dose RBV arm (RD: 0.4% [95% CI: −2.4% to 3.2%]). During the first 6 weeks of treatment, 6.1% and 6.5% of the patients receiving weight-based and flat-dose RBV had anemia (RD: −0.4% [95% CI: −3.9% to 4.7%]). Furthermore, 20.2% and 15.0% of the patients receiving weight-based and flat-dose RBV had anemia (RD: 5.2% [95% CI: −1.5% to 11.9%]) between weeks 6 and 16 of treatment.

## Discussion

Our study demonstrated that Peg-IFN alfa-2a plus weight-based or flat-dose RBV for 16 week provided high SVR_24_ rates in HCV-2 rapid responders. The SVR_24_ rates in our patients (91.9% to 93.5%) were greater than those in HCV-2 slow responders receiving 24–48 weeks of Peg-IFN plus RBV therapy (46.2% to 52.6% for 24 weeks; 71.4% for 36 weeks; 68.4% to 77.8% for 48 weeks), suggesting that the early viral kinetics play an important role in determining the anti-viral responses in these patients^25^,^26^,^27^. Furthermore we also showed that treatment with Peg-IFN alfa-2a plus weight-based RBV provided a comparable SVR_24_ rate (93.5% versus 91.9%) and treatment-related withdrawal rate (2.8% versus 2.4%) to treatment with Peg-IFN alfa-2a plus flat-dose RBV, suggesting HCV-2 rapid responders may receive Peg-IFN plus flat-dose RBV for 16 weeks to achieve high a SVR rate and a low AE rate. While the SVR_24_ rates were similar in females in weight-based and flat-dose RBV arms (91.3% versus 97.1%), the SVR_24_ rate was greater in males receiving Peg-IFN alfa-2a plus weight-based RBV than that in males receiving Peg-IFN alfa-2a plus flat-dose RBV (95.1% versus 88.0%).

Although the SVR_24_ rates between weight-based and flat-dose RBV arms were similar stratified by baseline viral load, subgenotype, IL-28B genotype, age, weight, APRI score, RBV dosage or 80/80/80 rule, males in weight-based RBV arm achieved a significant greater SVR rate than those in flat-based RBV arm. We further examined the possible factors for the gender effects. The mean dosage of RBV in weight-based RBV arm was significantly greater than flat-dose RBV arm, regardless of gender. When we stratified the RBV dosage at a cut-off value of 13 mg/kg/day, we revealed that males with a RBV dosage of ≥13 mg/kg/day had a greater SVR_24_ rate than those with a dosage of <13 mg/kg/day, but the SVR_24_ rates in females were similar whether they received a RBV dosage of ≥13 mg/kg/day or not. Manns *et al.* demonstrated that the SVR_24_ rate tended to increase in HCV-infected patients if they received a RBV dosage of ≥13 mg/kg/day^5^. Our study implied that although males who achieved RVR after induction therapy with Peg-IFN alfa-2a plus weight-based RBV, a sufficiently high dosage of RBV (≥13 mg/kg/day) should be maintained to secure a high SVR rate if they received a truncated treatment duration for 16 weeks. In females who achieved RVR after Peg-IFN alfa-2a plus weight-based RBV induction therapy, flat-dose RBV therapy may be preferred at treatment weeks 6–16 because it did not compromise the SVR_24_ rate and may lessen the severity of RBV-induced anemia.

With regard to safety, treatment by Peg-IFN alfa-2a plus RBV for 16 weeks showed low rates of serious AE and treatment withdrawal in weight-based and flat-dose RBV arms. Except for the tendency of clinically significant anemia at treatment weeks 6–16 in weight-based RBV arm, the constitutional symptoms and the other laboratory abnormalities of interest were comparable between the two arms. However, the proportions of severe anemia (Hb < 8.5 g/dL) which warranted temporary RBV discontinuation were low (6.9% and 6.1%) in weight-based and flat-dose RBV arms, respectively, implying that by careful monitoring the on-treatment hemoglobin levels and titrating the RBV dosages, most patients receiving weight-based or flat-dose RBV may safely complete treatment.

Our study had several limitations. First, our patients were East Asians, and the results should be validated in patients of different ancestry. Second, with the introduction of sofosbuvir-base therapies which confers excellent treatment responses, Peg-IFN plus RBV therapy may not be listed in the first-line therapy in Western countries. Despite the potentially higher on-treatment AE rates by Peg-IFN plus RBV therapy, our study also showed that HCV-2 rapid responders may also achieve high SVR rates by 16 weeks of therapy. The use of our strategies may provide a practical guide of IFN-eligible HCV-2 patients in resource-limited countries for sofosbuvir-based therapy^28^.

In conclusion, Peg-IFN alfa-2a plus weight-based or flat-dose RBV for 16 weeks provides comparable SVR rates for treatment-naïve HCV-2 patients with RVR. However, males should receive Peg-IFN alfa-2a plus weight-based RBV to provide a high SVR rate.

## Materials and Methods

### Patients

Treatment-naïve Taiwanese HCV-2 patients who were old than ≥18 years who had serum alanine aminotransferase (ALT) level ≥upper limit of normal (ULN) were consecutively enrolled between 2007 and 2013 in the Tailored Regimens of Peginterferon alfa-2a and Ribavirin for Genotype 2 Chronic Hepatitis C Patients (TARGET-2) trial. Chronic HCV infection was defined as documentation of anti-HCV antibody (Abbott HCV EIA 3.0, Abbott Laboratories, Abbott Park, Illinois, USA) and HCV RNA (Cobas TaqMan HCV Test v2.0, Roche Diagnostics GmbH, Mannheim, Germany, limit of detection: 15 IU/mL) for ≥6 months. The HCV genotyping was tested by reverse hybridization assay (Versant HCV Genotype 2.0 assay, Siemens Healthcare Diagnostics, Illinois, USA)^29^.

The exclusion criteria of the study were as follows: hemoglobin levels <13 g/dL for men or <12 g/dL for women, neutrophil count <1.5 × 10^9^ cells/L, platelet count < 90 × 10^9^ cells/L, hepatitis B virus (HBV) or human immunodeficiency virus (HIV) co-infection, HCV infection other than genotype 2, alcohol consumption >20 g/day, serum albumin level <35 g/L, serum bilirubin level ≥1.5 times ULN, serum AST or ALT level ≥10 times ULN, serum creatinine level ≥1.5 times ULN, Child-Puge grade B or C cirrhosis, history of autoimmune liver diseases or neoplastic diseases, concurrent immunosuppressive therapy, pregnancy, poorly controlled systemic illness or unwilling to receive birth control during the study period.

The study was approved by Taiwan Joint Institutional Review Board. All study procedures were conducted in accordance with the principles of Declaration of Helsinki and the International Conference on Harmonization for Good Clinical Practice. Patients who were willing to join the study provided written informed consent before enrollment.

### Study Design

**Figure 2:**
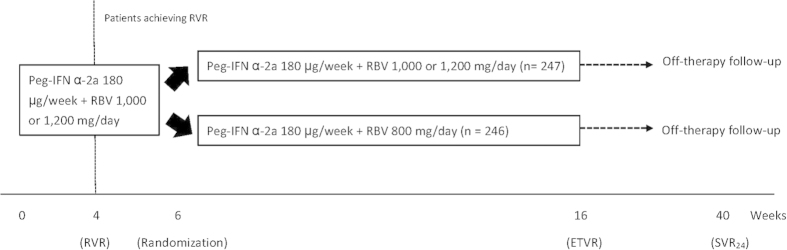
Study Design. Peg-IFN: peginterferon, RBV: ribavirin; RVR: rapid virologic response, ETVR: end-of-treatment virologic response, SVR_24_: sustained virologic response 24 weeks off-therapy.

This was a multicenter randomized controlled trial. All patients who were eligible to participate in this trial received induction therapy of Peg-IFN alfa-2a 180 μg/week (Pegasys, Hoffman-LaRoche, Basel, Switzerland) plus weight-based RBV 1,000 or 1,200 mg/day (Copegus, Hoffman-LaRoche, Basel, Switzerland; cut-off body weight: 75 kg) for 6 weeks. The viral responses at week 4 of treatment were evaluated for all patients. At week 6 of treatment, patients who achieved rapid virologic response (RVR), defined as undetectable serum HCV RNA at week 4 of treatment, were randomly assigned as 1:1 ratio to receive Peg-IFN alfa-2a 180 μg/week plus weight-based (1,000 or 1,200 mg/day) or flat-dose (800 mg/day) RBV for additional 10 weeks (Fig. 2). Randomization code was computer-generated in blocks of 4 and secured by an independent assistant. In addition, all patients were centrally allocated to the assigned treatment.

Baseline demographic data, hemoglobin level, neutrophil count, and platelet count, serum albumin, serum bilirubin, serum AST, serum ALT, serum creatinine, anti-HCV, HBsAg, anti-HIV, HCV RNA, HCV genotype, and IL-28B rs8099917 genotypes (ABI TaqMan allelic discrimination kit and ABI7900HT Sequence Detection System, Applied Biosystems, Life Technologies Corporation, Grand Island, New York, USA) were evaluated. The hepatic fibrosis was staged by APRI^30^. Low baseline viral load was defined as a level of <800,000 IU/mL, whereas high baseline viral load was defined as a level of ≥800,000 IU/mL^11^. IL-28B rs8099917 TT genotype was defined as favorable genotype, and GT/GG genotype was defined as unfavorable genotype, respectively^31^,^32^,^33^. An APRI score of >1.50 and >2.00 denoted significant hepatic fibrosis (≥F2 by Metavir score) and cirrhosis (F4 by Metavir score), respectively^34^.

### Efficacy

All patients received treatment for 16 weeks and the off-therapy follow-up for 24 weeks. Serum HCV RNA levels were assessed at weeks 4 and 16 of treatment, and at week 24 after the cessation of therapy. The ETVR and the SVR_24_ were defined as previously described^11^. The ETVR was assessed for all patients who early discontinued treatment at the treatment discontinuation.

The primary endpoint was SVR_24_, defined as undetectable serum HCV RNA 24 weeks after the cessation of therapy. Patients with viral breakthrough at the end-of-treatment were considered failure to achieve SVR_24_, regardless of the end of follow-up HCV RNA data. Patients who lacked the end of follow-up data to assess SVR_24_ or who relapsed after the cessation of treatment were also considered failure to achieve SVR_24_. Sensitivity analyses were done for the primary endpoint.

### Safety

During the study period, the safety profiles were reported by a prespecified checklist to assess the severity and the causality of the AEs. The grades of all AEs were assessed by the Common Terminology Criteria for Adverse Events (CTCAE), version 3.0. If the patients had serious AEs, missed he allocated treatment for >4 consecutive weeks, or subjectively stopped treatment, they were considered withdrawn from the study.

Based on the severity of the constitutional AEs, the dosage of Peg-IFN alfa-2a and RBV was reduced in a stepwise fashion of 45 μg/week and 200 mg/day as tolerated. With regard to hematological toxicity, Peg-IFN alfa-2a was reduced from 180 μg/week to 90 μg/week if the neutrophil count was <0.75 × 10^9^ cells/L or the platelet count was <50 × 10^9^ cells/L, and Peg-IFN alfa-2a was stopped if the neutrophil count was <0.50 × 10^9^ cells/L or the platelet count was <25 × 10^9^ cells/L. RBV was reduced in a stepwise fashion of 200 mg/day if the hemoglobin level was <10 g/dL, and RBV was stopped if the hemoglobin level was <8.5 g/dL. The erythropoiesis-stimulating agent (ESA) was not permitted during the treatment. Blood transfusion was allowed if the patients developed serious hematological AEs. Peg-IFN or RBV was allowed to be reinitiated or increased in dosage as tolerated if the constitutional or laboratory AEs improved following treatment cessation or dosage reduction.

### Statistical Analyses

Analysis of the data was performed by Statistical Program for Social Sciences (SPSS 17.0; SPSS Inc., Chicago, Illinois, USA). Based on the assumption that the SVR_24_ rate was 86% for patients assigned to receive Peg-IFN plus flat-dose RBV, we estimated that 488 patients would provide 90% power to detect an absolute increase of 8% or more in SVR_24_ for patients assigned to receive Peg-IFN plus weight-based RBV (2-sided α = 0.05)^12^,^18^. Values were expressed as mean (standard deviation, SD) or percentage when appropriate.

The on-treatment and off-therapy viral response rates between weight-based and flat-dose RBV arms were compared by RD. The *P* values for RD were assessed by the Wald asymptotic test. The subgroup analyses for the factors of interest to predict SVR_24_, including baseline viral load, HCV subgenotype, IL-28B rs8099917 genotype, age, sex, body weight, APRI score, RBV dosage, and 80/80/80 rule were compared by RD^35^. The interactions between the prespecified factors and the treatment arms were evaluated by the stratified Mantel-Haenszel test. The dosage of RBV associated with SVR was compared by chi-square with Fisher’s exact test. All statistical tests were two-tailed and the results were considered to be statistically significant when a *P* value was <0.05.

## Additional Information

**How to cite this article**: Liu, C.-H. *et al.* Peginterferon alfa-2a plus Weight-Based or Flat-Dose Ribavirin for Treatment-Naïve Hepatitis C Virus Genotype 2 Rapid Responders: A Randomized Trial. *Sci. Rep.* **5**, 15255; doi: 10.1038/srep15255 (2015).

## Figures and Tables

**Table 1:** Baseline Patient Characteristics*.

Characteristics	Peg-IFN plus weight-based RBV, N = 247	Peg-IFN plus flat-dose RBV, N = 246
Mean age (SD), y	55 (11)	56 (11)
Age >50 y	163 (66)	168 (68)
Male	143 (58)	142 (58)
Mean weight (SD), kg	66 (11)	65 (10)
Male	70 (8)	69 (10)
Female	60 (9)	61 (8)
Weight ≥75 kg	55 (22.3)	52 (21.1)
Mean BMI (SD), kg/m^2^	25.2 (3.3)	25.3 (3.4)
Mean hemoglobin level (SD), g/dL	14.4 (1.4)	14.2 (1.4)
Mean white cell count (SD), 10^9^cells/L	3.1 (1.1)	3.0 (1.1)
Mean neutrophil count (SD), 10^9^cells/L	176 (48)	173 (50)
Mean platelet count (SD), 10^9^cells/L	2.2 (1.4)	2.3 (1.3)
Mean albumin level (SD), g/dL	4.2 (0.4)	4.2 (0.9)
Mean total bilirubin level (SD), mg/dL	0.9 (0.3)	1 (0.4)
Mean AST quotient (SD), ULN	2.2 (1.4)	2.3 (1.3)
Mean ALT quotient (SD), ULN	3.4 (2.4)	3.4 (2.2)
Mean APRI score (SD)	1.4 (1.2)	1.5 (1.2)
APRI score		
≤1.50	158 (64.0)	149 (60.6)
1.51–2.0	34 (13.7)	35 (14.2)
>2.00	55 (22.3)	62 (25.2)
Mean HCV RNA level (SD), log_10_ IU/mL	5.4 (0.8)	5.3 (0.8)
HCV RNA level		
≤800,000 IU/mL	195 (79)	193 (78)
>800,000 IU/mL	52 (21)	53 (22)
Subgenotype		
2a	185 (75)	185 (75)
2b	52 (21)	46 (19)
2a + 2b	10 (4)	15 (6)
IL-28B rs8099917 genotype†		
TT	203 (86)	202 (88)
GT and GG	32 (14)	28 (12)

Peg-IFN: peginterferon, RBV: ribavirin, SD: standard deviation, BMI: body mass index, AST: aspartate aminotransferase, ALT: alanine aminotransferase, ULN: upper limit of normal, APRI: aspartate aminotransferase to platelet ratio index, HCV: hepatitis C virus, IU: international unit, IL: interleukin.

^*^Values are numbers (percentages) unless otherwise indicated.

^†^Available number of patients (%) for analysis: 235 (95%) and 230 (93%) in weight-based RBV and flat-dose RBV arms, respectively.

**Table 2:** On-treatment and Off-therapy Virologic Responses.

Variable	eg-IFN plus weight-based RBV, n/N (%)	Peg-IFN plus flat-dose RBV, n/N (%)	RD (95% CI)	*P* value*
On-treatment virologic response				
ETVR	247/247 (100.0)	245/246 (99.6)	0.4 (−0.7 to 1.5)	0.50
Virologic outcome				
SVR_24_†	231/247 (93.5)	226/246 (91.9)	1.7 (−2.9 to 6.2)	0.49
Non-SVR_24_	16/247 (6.5)	20/246 (8.1)		
Relapse	10/247 (4.0)	10/246 (4.1)		
Null-response	0	0		
Viral breakthrough‡	0	1/246 (0.4)		
Undetermined§	6/247 (2.4)	9/246 (3.7)		

Peg-IFN: peginterferon, RBV: ribavirin, RD; risk reduction, EVR: early virologic response, ETVR: end-of-treatment virologic response, SVR: sustained virologic response, CI: confidence interval.

^*^P values were obtained by Wald asymptotic test.

^†^Patients who were lost to 24-week follow-up, were null-responsive to treatment, or had viral breakthrough or relapsed after treatment were considered failure to achieve SVR_24_.

^‡^One patient in flat-dose RBV arm had viral breakthrough at week 16 of treatment.

^§^All patients lost to 24-week off-therapy follow-up, and all had undetectable HCV RNA level at the time of treatment discontinuation. Weight-based RBV arm: 5 completed 16 weeks of treatment, and 1 prematurely discontinued treatment at week 8 due to serious adverse event. Flat-dose RBV arm: 8 completed 16 weeks of treatment, and 1 prematurely discontinued treatment at week 12 due to adverse event.

**Table 3:** Subgroup Analyses of Prespecified Factors for SVR_24_.

Variable	Peg-IFN plus weight-based RBV, n/N (%)	Peg-IFN plus flat-dose RBV, n/N (%)	RD (95% CI)	*P*value for interaction*
Baseline viral load				0.33
≤800,000 IU/mL	191/195 (97.9)	185/193 (95.9)	2.1 (−1.4 to 5.5)	
>800,000 IU/mL	40/52 (76.9)	41/53 (77.4)	−0.4 (−16.5 to 15.6)	
Subgenotype				0.61
2a	173/185 (93.5)	170/185 (91.9)	1.6 (−3.7 to 6.9)	
2b	49/52 (94.2)	42/46 (91.3)	2.9 (−7.4 to 13.2)	
2a + 2b	9/10 (90.0)	14/15 (93.3)	3.3 (−19.1 to 25.8)	
IL-28B rs8099917 genotype†				0.99
TT	189/203 (93.1)	183/202 (90.6)	2.5 (−2.8 to 7.8)	
GT and GG	30/32 (93.8)	28/28 (100)	−6.3 (−16.5 to 3.9)	
Age				0.18
≤50 y	79/84 (94.0)	68/78 (87.2)	6.9 (−2.1 to 15.9)	
>50 y	152/163 (93.3)	158/168 (94.0)	−0.8 (−6.1 to 4.5)	
Sex				0.01
Female	95/104 (91.3)	101/104 (97.1)	−5.8 (−12.1 to 0.5)	
Male	136/143 (95.1)	125/142 (88.0)	7.1 (0.7 to 13.6)	
Weight				0.71
<75 kg	181/192 (94.3)	181/194 (93.3)	1.0 (−3.8 to 5.8)	
≥75 kg	50/55 (90.9)	45/52 (86.5)	4.4 (−7.6 to 16.4)	
APRI score				0.11
≤1.50	145/158 (91.8)	136/149 (91.3)	0.5 (−5.7 to 6.7)	
1.51–2.00	31/34 (91.8)	34/35 (97.1)	−6.0 (−17.0 to 5.1)	
>2.00	55/55 (100)	56/62 (90.3)	9.7 (−1.8 to 17.6)	
RBV dosage‡				0.88
<13 mg/kg/day	34/40 (85.0)	114/130 (87.7)	−2.7 (−15.1 to 9.7)	
≥13 mg/kg/day	197/207 (95.2)	112/116 (96.6)	−1.4 (−5.8 to 3.0)	
Meet 80/80/80 rule§				0.64
Yes	203/216 (94.0)	204/222 (91.9)	2.1 (−2.7 to 6.9)	
No	28/31 (90.3)	22/24 (91.7)	−1.3 (−16.5 to 13.8)	

Peg-IFN: peginterferon, RBV: ribavirin, RD: risk reduction, CI: confidence interval, IL: interleukin, BMI: body mass index, APRI: aspartate aminotransferase to platelet ratio index, RBV: ribavirin.

^*^The interaction for the prespecified factors was compared by stratified Mantel-Haenszel test.

^†^Available number of patients (%) for analysis: 235 (95%) and 230 (93%) in weight-based RBV and flat-dose RBV arms, respectively.

^‡^The daily RBV dosage was calculated by total exposure of ribavirin divided by the actual duration of treatment and the baseline body weight.

^§^Denotes patients who received ≥80% of both Peg-IFN alfa-2a and RBV doses for ≥80% of the expected duration of therapy.

**Table 4:** Daily Ribavirin Dosage and the SVR_24_ Rates in Male and Female Patients.

Gender	Ribavirin dosage		*P* value
	<13 mg/kg/day	≥13 mg/kg/day	
	SVR_24_, n/N (%)	SVR_24_, n/N (%)	
Female	46/49 (93.9)	150/159 (94.3)	0.99
Male	102/121 (84.3)	159/164 (97.0)	<0.001
Total	148/170 (87.1)	309/323 (95.7)	0.001

**Table 5:** Adverse Events, Dose reduction and Treatment Discontinuation in Treated Patients.*

Parameter	Peg-IFN plus weight-based RBV, N = 247	Peg-IFN plus flat-dose RBV, N = 246
Serious AEs		
All†	2 (0.8)	1 (0.4)
Death	0 (0)	0 (0)
Treatment-related	2 (0.8)	1 (0.4)
Treatment withdrawal due to AEs	7 (2.8)	6 (2.4)
Dose reduction to AEs		
Peginterferon	38 (15.3)	36 (14.6)
Ribavirin	59 (23.9)	49 (19.9)
Constitutional AEs		
Flu-like symptoms	71 (28.7)	69 (28.0)
Fatigue	145 (58.7)	146 (59.3)
Headache	73 (29.6)	70 (28.5)
Insomnia	90 (36.4)	88 (35.8)
Irritability	24 (9.7)	25 (10.2)
Depression	21 (8.5)	19 (7.7)
Anorexia	65 (26.3)	62 (25.2)
Diarrhea	29 (11.7)	27 (11.0)
Constipation	20 (8.1)	18 (7.3)
Cough	39 (15.8)	37 (15.0)
Dermatitis	67 (27.1)	64 (26.0)
Injection site reaction	36 (14.6)	33 (13.4)
Hair loss/alopecia	46 (18.6)	45 (18.3)
Laboratory AEs‡		
Anemia (week 1–6)§	15 (6.1)	16 (6.5)
Hemoglobin level: 8.5–9.9 g/dL	11 (4.5)	13 (5.3)
Hemoglobin level:<8.5 g/dL	4 (1.6)	3 (1.2)
Anemia (week 6 to end-of-treatment)§	50 (20.2)	37 (15.0)
Hemoglobin level: 8.5–9.9 g/dL	33 (13.4)	22 (8.9)
Hemoglobin level: <8.5g/dL	17 (6.9)	15 (6.1)
Neutropenia	26 (10.5)	24 (9.8)
Neutrophil count: 0.500–0.749 × 10^9^ cells/L	21 (8.5)	20 (8.1)
Neutrophil count: <0.500 × 10^9^ cells/L	5 (2.0)	4 (1.6)
Thrombocytopenia	25 (10.1)	23 (9.3)
Platelet count: 25–49 × 10^9^ cells/L	22 (8.9)	21 (9.1)
Platelet count: <25 × 10^9^ cells/L	3 (1.2)	2 (0.8)
ALT elevation		
>2 times ULN	35 (14.2)	34 (13.8)
>5 times ULN	5 (2.0)	4 (1.6)
Total bilirubin elevation||		
>2 mg/dL	11 (4.5)	9 (3.7)
>5 mg/dL	0 (0.0)	0 (0.0)

Peg-IFN: peginterferon, RBV: ribavirin, AE: adverse event, ALT: alanine aminotransferase, ULN: upper limit of normal.

^*^Values are numbers (percentages).

^†^Weight-based RBV arm: major depression at week 8 of treatment, and anxiety disorder at week 7 of treatment, respectively. Flat-dose RBV arm: peripheral edema at week 14 of treatment. All of the 3 patients were considered treatment-related.

^‡^The grading of the laboratory AEs was shown for patients with the on-treatment nadir level.

^§^Anemia was defined as a nadir hemoglobin level <10.0 g/dL.

^||^No patient with total bilirubin elevation had concomitant ALT elevation >5 times ULN.
